# Cross-Project Defect Prediction Based on Two-Phase Feature Importance Amplification

**DOI:** 10.1155/2022/2320447

**Published:** 2022-04-18

**Authors:** Ying Xing, Wanting Lin, Xueyan Lin, Bin Yang, Zhou Tan

**Affiliations:** ^1^School of Artificial Intelligence, Beijing University of Posts and Telecommunications, 100876 Beijing, China; ^2^Du Xiaoman (Beijing) Science Technology Co., Ltd., 100000 Beijing, China

## Abstract

As the typical application of computational intelligence in software engineering, cross-project defect prediction (CPDP) uses labeled data from other projects (source projects) for building models to predict the defects in the current projects (target projects), helping testers quickly locate the defective modules. But class imbalance and different data distribution among projects make CPDP a challenging topic. To address the above two problems, we propose a two-phase feature importance amplification (TFIA) CPDP model in this paper which can solve these two problems from domain adaptation phase and classification phase. In the domain adaptation phase, the differences in data distribution among projects are reduced by filtering both source and target projects, and the correlation-based feature selection with greedy best-first search amplifies the importance of features with strong feature-class correlation. In the classification phase, Random Forest works as the classifier to further amplify the importance of highly correlated features and establish a model which is sensitive to highly correlated features. We conducted both ablation experiments and comparison experiments on the widely used AEEEM database. Experimental results show that TFIA can yield significant improvement on CPDP. And the performance of TFIA CPDP model in all experiments is stable and efficient, which lays a solid foundation for its further application in practical engineering.

## 1. Introduction

Software defects may cause unexpected disasters in the application scenarios, threatening the security of software and even people's lives [1]. During the whole life cycle of software, defects that are found in the later period will cost more than those found in the earlier period to be repaired. Testers should find out the defects in time, helping developers fix them as soon as possible [2]. But focusing on all modules of the software will cost a large amount of time and manpower, which goes against our original intention and makes preidentification of potentially defective modules an urgent issue [3, 4].

Software defect prediction (SDP) is a method that can assist testers in quickly identifying potentially defective modules [5] so as to reduce the time spent by testers on troubleshooting and save testing costs as well [4]. The typical SDP usually uses historical data of software to build prediction models for defect pattern recognition in new releases of the modules from the projects [3, 6, 7]. As the brand-new projects lack historical data, especially the historical data with defective or clean labels, SDP is unable to work well on them. To solve this problem, researchers propose a method called cross-project software defect prediction (CPDP). CPDP builds prediction models with historical data from other projects (source projects) and predicts the current projects (target projects) [8, 9].

Although CPDP focuses on the problem of lack of historical data, there are still two major problems to be solved. Due to a variety of factors such as project functionality and developers' habits, data distribution varies greatly from project to project. Differences in data distribution between projects are evident even when the same metrics are used to evaluate projects [10], which makes CPDP models built on source projects ineffective on the target project [11]. Therefore, how to reduce the differences in data distribution between source and target projects is important to the performance of CPDP models [12, 13]. On the other hand, the number of the modules with defects is often much smaller than that of the modules without defects, which is called class imbalance problem [14]. The class imbalance problem can also affect the performance of CPDP models, because they may have a preference for the majority when classifying [15, 16].

To address the above problems, in this paper we propose a two-phase feature importance amplification (TFIA) CPDP model. Specifically, TFIA divides CPDP into two phases. In the domain adaptation phase, TFIA reduces differences in data distribution among source and target projects. After adding a certain proportion of samples from target projects to the source projects, the correlation-based feature selection method with a greedy best-first search strategy is proposed to amplify the importance of features with high feature-class correlation. The subset made up of the final selected items is used to filter the source and target projects for the purpose of removing redundant features as well as interfering items and reducing the dimensionality of the data. In the classification phase, resampling is conducted on the source project's data to make the numbers of clean and defective samples balanced. And an integrated learning method, Random Forest, is adopted to build the prediction model. The reason for adopting Random Forest is that integrated learning methods have shown their advantages in solving the class imbalanced problem [17]. In our case, the selected Random Forest uses a random sampling method; thus the trained model has small variance and high generalization ability, which can improve and effectively solve the class imbalance problem. Random Forest evaluates the relationship between features and classes during the training process [18], resulting in a ranking of feature-class correlation [19], further amplifying the importance of features with strong feature-class correlation for defect recognition.

In summary, our contributions lie in twofold.

1. We propose a two-phase feature importance amplification CPDP model (TFIA). TFIA reduces the effect of data distribution and class imbalance problems in domain adaptation phase and classification phase.
2. We conducted experiments on the AEEEM database to verify the validity of the method. In addition, we performed a number of ablation experiments to carefully analyse and dissect the detailed components of our method. And we also conducted comparison experiments to compare TFIA with other methods.

## 2. Related Work

CPDP usually includes two phases, called domain adaptation and classification. Domain adaptation is mainly addressed by data processing methods, such as data migration and feature selection. Classification is mainly handled by machine learning classifiers [20]. The researches on domain adaptation address the difference in data distribution between the source and target projects, while the researches on classification methods improve the prediction performance from the perspective of algorithms. And also some researchers consider hybrid methods.

### 2.1. Domain Adaptation Phase

Turhan et al. [21] proposed Burak filter based on k-means clustering method. Burak filter calculated the Euclidean distance between all samples in the source project datasets. And for each sample in the target project datasets, they selected the *k* samples with the smallest Euclidean distance as training samples. Finally, the Naive Bayes method was used to predict the processed dataset. Burak filter improves the performance of CPDP model by filtering the samples of the source project datasets based on the target project datasets. Peters et al. [22] proposed Peters filter based on the source project datasets. For the samples in the source project datasets, the sample which was the closest to it in the target project datasets was selected by comparing the Euclidean distance. Then they labeled that sample. The sample of source project datasets with the closest distance to the labeled sample was selected as the training data in the prediction process. Pan et al. [23] proposed the TCA, which mapped the features of the source and target project to the latent space that makes them most similar, reducing the effect of differences in data distribution. Finally, Logistic Regression was used for prediction. He et al. [24] simplified the training set by TDSelector method and then classified it by Logistic Regression. Sun et al. [25] proposed a near-some source project selection by collaborative filtering (CFPS) method to filter source items, which has good results using SMO and Random Forest as classifiers. Alsawalqah et al. [26] proposed a SMOTE-ensemble method to optimize for class imbalance problems at the data level and algorithm level.

### 2.2. Hybrid Method

Yuan et al. [27] used TrAdaBoost to determine weights for samples based on Burak filter and used weighted support vector machines to build the model to improve the CPDP model. Chao et al. [28] proposed a two-phase CPDP method called TPTL, using a source project estimator to select source projects with similar data distribution as the target project and using two improved TCA + to construct models for prediction. Cong [29] proposed a DA-KTSVMO method using kernel twin support vector machines to improve the data distribution and using a quantum particle swarm optimization algorithm to optimize the method for prediction. Zhang et al. investigated seven composite algorithms; they believed that composite algorithms can improve the performance of CPDP models [30]. The method proposed by Chen et al. combines the data gravitation method and TrAdaBoost to reduce the effects of class imbalance in the source project [31]. Xu et al. proposed a multisource TrAdaBoost Algorithm. The proposed method uses semisupervised high-density-based clustering and a small amount of labeled target item data to obtain a large amount of labeled source item data. When using TrAdaBoost for integration, only the base classifier trained from the source item data most relevant to the target item is selected. In the process of training, this method can ensure that the knowledge transferred is most relevant to the target project but ignores the influence of other source project data on the target project defect prediction model [32].

## 3. Methodology

In this section, we first introduce the framework of our proposed method TFIA. Then we show the details of important steps in our proposed method.

### 3.1. Framework of TFIA

**Figure 1:**
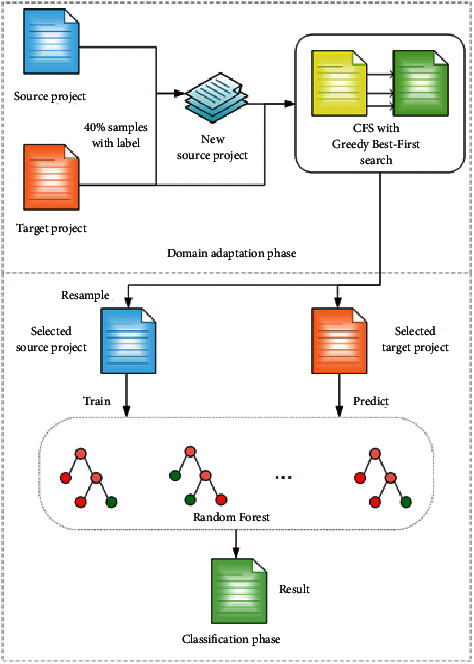
Flowchart of TFIA.

Figure 1 is the flowchart of TFIA. TFIA adds a certain proportion of labeled samples from the target project datasets to the source project datasets, uses a correlation-based feature selection method, and searches for a subset of features using a heuristic greedy best-first search strategy. Then TFIA filters the feature of source and target projects, samples the source projects datasets with resampling, uses Random Forest on the source project datasets for training, and finally predicts the target project datasets.

TFIA reduces the difference in data distribution between source and target projects from the feature perspective and sample perspective, respectively. TFIA enhances the importance of features with strong class-feature correlation in model training. A resample approach is used to deal with class imbalance problems and reduce the impact generated by class imbalance problem. TFIA uses Random Forest as a classifier to train models with small variance and strong generalization ability to improve the accuracy of CPDP model for identifying defective modules.

In TFIA, we use Relief to evaluate the correlation of feature-feature and feature-class in TFIA. Relief[33] is a feature weighting algorithm that is sensitive to feature interactions. Relief of feature *f* can be formulated as

(1) Relieff=Gini'×∑fv∈fpfv21−∑c∈Cpc2∑c∈Cpc2.

p is the probability, *C* is the class variable, *C*={defective, clean}, *f*_*v*_ is the certain value of the sample's feature *f*, and *Gini*' is another attribute quality measure that can be calculated by

(2) $$\begin{array}{c} {Gini}^{\prime }=\left[\underset{c\in C}{\sum }p\left(c\right)\left(1-p\left(c\right)\right)\right]-\underset{f_{v}\in f}{\sum }\left(\frac{p{\left(f_{v}\right)}^{2}}{\sum^{}f_{v}\in f}p{\left(f_{v}\right)}^{2\sum_{c\in C}p\left(cf_{v}\right)\left(1-p\left(cf_{v}\right)\right)}\right). \end{array}$$

We use Relief to calculate correlation of feature-feature and feature-class in Section 3.2 and Section 3.3.

### 3.2. Domain Adaptation Phase

In order to amplify the role of features with strong feature-class correlation in the classifier and to deemphasize features with strong feature-feature correlation, we decide to select the features. In the feature selection phase, we use the filtering method called correlation-based feature selection (CFS) [34]. In this paper, the heuristic search strategy used by CFS is greedy best-first search [35]. CFS evaluates the value of a subset of features by considering the individual predictive ability of each feature and the degree of redundancy between them [36].

**Figure 2:**
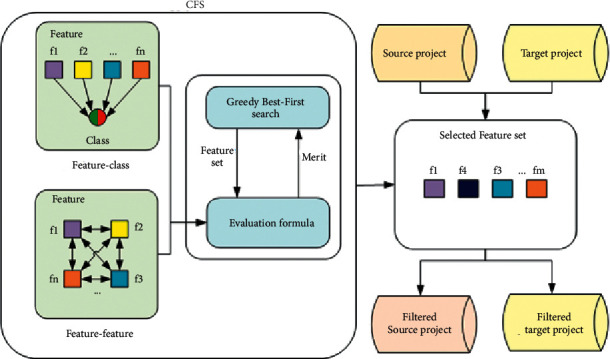
Flowchart of CFS.

As shown in Figure 2, CFS first computes the feature-class and feature-feature correlation matrices from the source project dataset and then searches the feature subset space using greedy best-first search.

To prevent the best-first search from exploring the entire feature subset search space, we follow the setting of [34] to impose a termination criterion. The search will terminate if five consecutive fully expanded subsets show no improvement over the current best subset. CFS filters features by a feature subset evaluation function, intending to find a subset of features that meet the conditions of low feature-feature correlation and strong feature-class correlation, thus sifting out redundant features [37]. The feature subset evaluation function is valued as the merit which can be calculated by

(3) $$\begin{array}{c} M_{S}=\frac{k\bar{r_{fc}}}{\sqrt{k+k\left(k-1\right)\bar{r_{ff}}}}, \end{array}$$

*M*_*S*_ is the value of the merit of feature subset with k selected features. $\bar{r_{fc}}$ is the average feature-class correlation and $\bar{r_{ff}}$ is the average feature-feature correlation. *r* is Relief in Section 3.1.

The algorithm is described as follows.

### 3.3. Classification Phase

Random Forest is an integrated learning pattern recognition method [19, 38]. It has been demonstrated that Random Forest has good performance in CPDP [27, 39, 40] due to its high tolerance to outliers and noise. Random Forest is also less prone to fitting characteristics [18].

**Figure 3:**
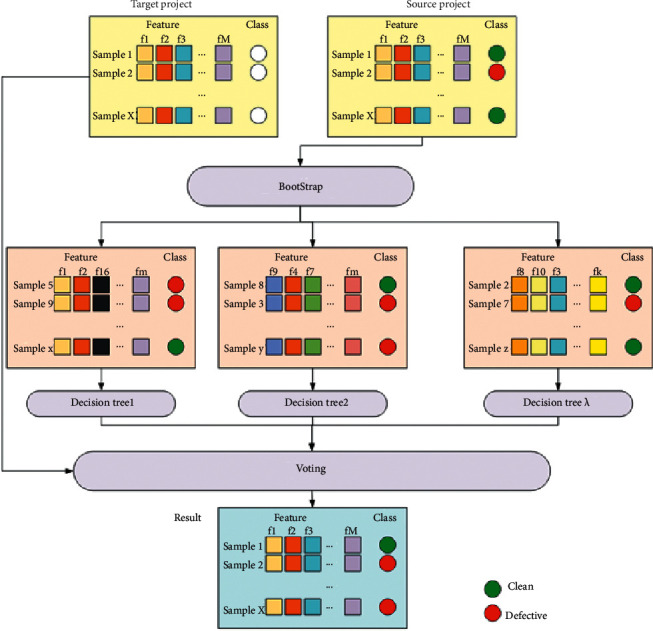
Flowchart of Random Forest.

Figure 3 shows the process of Random Forest in this paper. Random Forest uses a bootstrap method to randomly scrape multiple samples from the original samples, modeling a Decision Tree for each sample set. Then we give a comprehensive conclusion from the results of all decision trees. The voting decision process of Random Forest is shown by

(4) $$\begin{array}{c} H\left(x\right)\;=\text{majority vote}\left\{\underset{C}{\text{max}}\overset{k}{\underset{i=1}{\sum }}I\left(h_{i}\left(x\right)=C\right)\right\}, \end{array}$$

*H*(*x*) denotes the combined classification model, *h*_*i*_ denotes the single subject Decision Tree, *C* is the set of class labels, and *I*(·) is the indicative function.

There is a put-back from the source project dataset *D* processed in the domain adaptation phase to obtain a randomly selected subset *D*_*T*_*i*__ as the training set. And the sample size of the training dataset is the same as the original data.

This sampling method of Random Forest ensures the variability of the training set. For the sake of easy explanation, we assume that there are n samples in dataset *D*. The probability of each sample being picked is 1/*n*. Repeat it for n times, so the probability of each sample in *D* not being picked is (1 − 1/*n*)^*n*^. As n tends to infinity, $\underset{n⟶\infty }{\lim}{\left(1-1/n\right)}^{n}=0.368$. We can assume that 37% of the samples in *D* will not appear, which guarantees the variability of the training set. *λ* features are randomly selected from the source project dataset to construct a Decision Tree, and each node is based on (2).

In a single Decision Tree, the *Gini*' metric is calculated for each attribute and a variable with the minimum *Gini*′ metric is selected to split the current node. The Decision Tree is constructed by recursion until the stop criterion is reached.

The algorithm is described as follows.

## 4. Experimental Design

For our method TFIA, in this section, we raise four research questions and set up experiments to discuss and analyse each question.

### 4.1. Research Question

RQ1: Does the feature-level filtering approach proposed in this paper have any impact on the performance of the model?

In TFIA, we use filtering to reduce the data distribution difference in source and target projects datasets by CFS. Therefore we intend to analyse the role of filtering methods in the whole CPDP model.

RQ2 : In the classification phase, does the choice of classifier affect the overall performance?

In TFIA, feature-class correlation is also amplified in the classification phase as we use Random Forest as the classifier. Classifiers behave diversely on different types of data, so we want to investigate how other classifiers would perform on our model, such as the linear classifiers Logistic Regression and Support Vector Classification, the Bayesian formula-based classifier Naive Bayes, and the tree-based classifier Decision Tree.

RQ3: When features are transferred at the sample level, does the proportion of transfer affect the results?

In the domain adaptation phase of TFIA, a certain percentage of samples of the labeled target items are required. We want to analyse how different proportions of samples of labeled target items in the project affect the overall prediction performance.

RQ4 : Compared with the classical method and the latest research, does the method proposed in this paper improve CPDP model's performance?

Various methods have been proposed by researchers for the CPDP problem, such as the classical methods TCA [23], Peters filtering method [22], Burak filtering method [21], and the newer method ALTRA [27]. We seek to analyse how the performance obtained by TFIA differs from these methods.

### 4.2. Dataset

In this paper, we use the AEEEM database, a widely used dataset in the field of software defect prediction research. The AEEEM database was collected and compiled by D'Ambros et al. [41].  Table 1 shows the details of the AEEEM database, including the projects names, the projects types, numbers of modules, numbers of defective modules, and the ratio of defective modules.

### 4.3. Experimental Environment

All of our codes are written in *Python* 3.7. The GPU used for the experiments is NVIDIA TITAN V and the CPU is Intel I9-9920X. Classifications are realized by WEKA [42] with default parameters.

### 4.4. Performance Measure

The confusion matrix is used to store the correct and incorrect decisions made by the prediction model. The purpose of our defect prediction is to test out defective data. In this paper, we believe that a sample itself is defective, and the classifier also considers it defective, called True Positive (TP). The classification is False Negative (FN) if the classifier thinks it is clean. Similarly, if a sample does not have defects, the classifier classifies it as defective, called False Positive (FP); the classifier classifies it as free of defects, called True Negative (TN).

### 4.5. F1-Measure

F1-measure, also known as F-score, is a weighted summed average of Precision and Recall. Precision is the ratio of correctly predicted defective modules to all modules predicted to be defective, calculated as shown by

(5) $$\begin{array}{c} \text{Precision}=\frac{TP}{TP+FP}. \end{array}$$

Recall is the ratio of correctly predicted defective modules to all truly defective modules, calculated as shown in

(6) $$\begin{array}{c} \text{Recall}=\frac{TP}{TP+FN}. \end{array}$$

F1-measure is often used to evaluate the performance of a classification model. F1-measure can be calculated by

(7) $$\begin{array}{c} F1-\text{measure}=\frac{2*\text{Precision}*\text{Recall}}{\text{Precision}+\text{Recall}}=\frac{2TP}{2TP+FP+FN}. \end{array}$$

### 4.6. Area under the ROC Curve

Area under the ROC curve (AUC) is used to evaluate the degree of discrimination obtained by the model. The value of AUC ranges in [0, 1]. AUC for random prediction is 0.5. The advantage of AUC is that AUC is insensitive to decision thresholds such as precision and recall. The higher the AUC, the better the prediction.

### 4.7. Matthews Correlation Coefficient

Matthews correlation coefficient (MCC) is used in machine learning as a measure of binary (2-category) quality of classification, which is introduced from biochemistry by Brian W. Matthews in 1975 [43].

MCC takes into account true and false, positives and negatives, and is generally regarded as a balanced measure that can be used even if the classes are of very different sizes [44]. MCC is essentially a correlation coefficient between the observed and predicted binary classification. It returns a value between -1 and +1. MCC = +1 indicates a perfect prediction, MCC = 0 indicates that the model is not better than a random prediction, and MCC = -1 indicates a complete inconsistency between prediction and observation.

The formula for the MCC is shown by

(8) $$\begin{array}{c} MCC=\frac{TP\times TN-FP\times FN}{\sqrt{\left(TP+FP\right)\left(TP+FN\right)\left(TN+FP\right)\left(TN+FN\right)}}. \end{array}$$

## 5. Results and Discussion

In this section, we present the experimental results and give the answers to questions from Section 3.

### 5.1. Answer for RQ1

To answer RQ1, we designed two groups of experiments on the AEEEM database, where we formed a total of 20 source-target project pairs from the five projects, including EQ, JDT, ML, LC, and PDE. The first group of experiments performed no filtering on these 20 source-target project pairs. And the second group of experiments were filtered by CFS based on greedy best-first search strategy proposed in this paper. Both sets of experiments used Random Forest as the classifier. Both groups of experiments randomly added 40% of the target project data to the source project dataset and source project datasets were dealt with through resample.

Table 2 shows the results of the evaluation metrics obtained on 20 source-target project pairs without (Model 1) and with (Model 2) the filtering method proposed in this paper.

From Table 2, it can be observed that the overall effect of the prediction models trained from the data processed by the filtering method is significantly improved (the data in bold), with an average F1-measure improvement of about 156.54%, AUC improvement of about 45.74%, and MCC improvement of about 173.46%. Numerically, in these 20 source-target project pairs, the models with filtering all work better than those without filtering.

**Figure 4:**
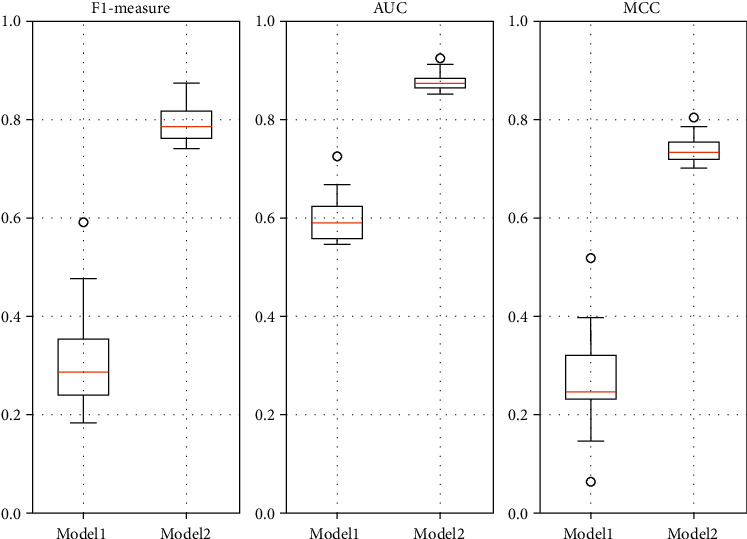
Boxplot of F1-measure, AUC, and MCC of model with filtering (Model 1) and model without filtering (Model 2).

Figure 4 is the boxplot of F1-measure, AUC, and MCC of model with filtering (Model 1) and model without filtering (Model 2) based on data from Table 2. As can be seen from Figure 4, the prediction models trained from the data processed by the filtering have higher numerical intervals in the overall distribution compared to the data without filtering. In terms of box size and endline length, the overall performance of the model after processing with the filtering also becomes less volatile and the performance is more stable. Therefore, we can conclude that the filtering method proposed in this paper is effective in improving the performance of the CPDP model on these datasets.

### 5.2. Answer for RQ2

To answer RQ2, 20 source-target projects from the AEEEM database were trained and validated using Naive Bayes (NB), Logistic Regression (LR), Decision Tree (DT), Support Vector Classification (SVC), and Random Forest (RF) as classifiers. Respectively, all the datasets were processed by the filtering method proposed in this paper. Every set of experiments randomly added 40% sample from the target project datasets to the source project datasets and the source project datasets were dealt with through resample.

Table 3 shows the F1-measure, AUC, and MCC of models with different classifications, including Naive Bayes (NB), Logistic Regression (LR), Decision Tree (DT), Support Vector Classification (SVC), and Random Forest (RF). The data in bold show the classification with the best performance in each set of experiments. The value of F1-measure shows that, on the data processed by our filtering method in this paper, RF has the best results, Decision Tree is the second best, and LR, NB, and SVC are more similar and differ from RF and DT. The situation is the same in AUC and MCC. This phenomenon could be caused by the idea that, in the first phase, our filtering method amplifies the proportion of features that have a strong correlation with the class. So it has better performance on DT and Decision Tree-based integrated method RF. The other three classifiers are relatively insensitive to the dataset of amplified features, so the results are worse than DT and RF.

**Figure 5:**
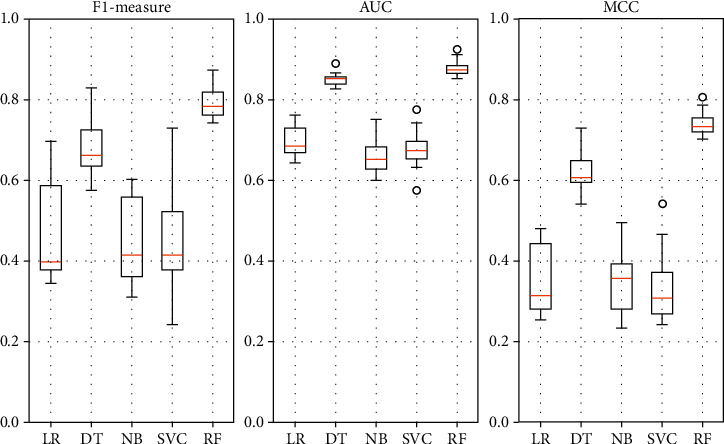
Boxplot of F1-measure, AUC, and MCC of models with different classifications, including Naive Bayes (NB), Logistic Regression (LR), Decision Tree (DT), Support Vector Classification (SVC), and Random Forest (RF).

Figure 5 is the boxplots of F1-measure, AUC, and MCC of models with different classifications, including Naive Bayes (NB), Logistic Regression (LR), Decision Tree (DT), Support Vector Classification (SVC), and Random Forest (RF). From Figure 5, it can be seen that the prediction models constructed with Random Forest and Decision Tree as classifiers achieve higher performance metrics and less overall volatility. This indicates that the choice of classifier has an impact on the performance of the prediction model. On the dataset processed by the filtering method in this paper, classifiers that are more sensitive to feature importance will have better results.

### 5.3. Answer for RQ3

To analyse the effect of the proportion of samples from target projects datasets added to the source projects datasets on the prediction results, we added different proportions of labeled target item samples to the source projects datasets in steps of 10% from 0% and trained with TFIA. Every source project dataset had been dealt with through resample.

Table 4 shows the average F1-measure, AUC, and MCC values of models added samples from target projects to the source projects at different proportions on 20 source-target project pairs.

**Figure 6:**
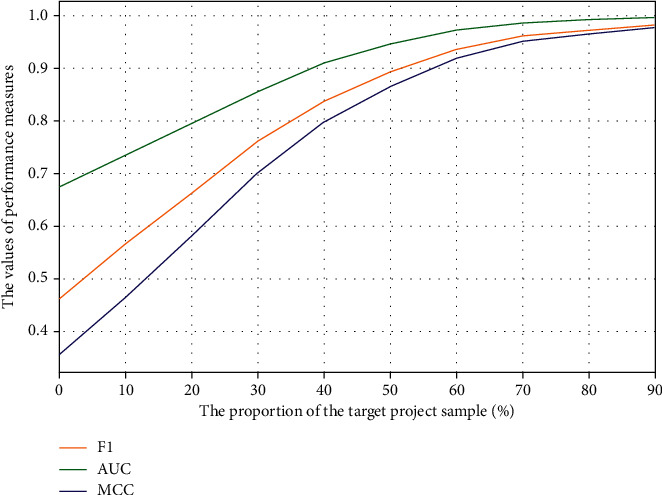
Average F1-measure, AUC, and MCC of TFIA using different proportions samples from target project datasets adding to source project datasets.

Then we smoothed the values to plot the graphs as Figure 6, from which we can see that the transformation of the three measures starts to become very small at around 70%, indicating that the model performance reaches its best at around 70%. But for the consideration of the actual prediction environment, it is not feasible to manually label the target projects samples at 70%, so we only consider the growth rate of indicators in this paper. It can be seen that the AUC starts to level off around 40%, and the improvement rates of F1-measure and MCC start to become smaller, so we believe that adding 40% of the labeled samples of the target projects to the source projects will have better results in these 20 source-target projects pairs.

### 5.4. Answer for RQ4

To answer RQ4, we used TCA, Burak filter, Peters filter, and ALTRA on 20 source-target project pairs in the AEEEM database for prediction and compared with our proposed method, respectively. To ensure the consistency of the experimental conditions, the source items in the dataset were randomly added to 40% samples of the target project datasets, and the source project datasets were dealt with through resample.

Table 5 shows the F1-measure, AUC, and MCC of ALTRA (Model 1), TCA (Model 2), Peters filter (Model 3), Burak filter (Model 4), and TFIA (Model 5).

As can be seen from the data in Table 5, compared with other methods, the proposed method in this paper shows significant improvements in F1-measure, AUC, and MCC. Taken as an example, the value of F1-measure is improved by about 126.62% over TCA, 98.64% over Burak filter, 86.46% over Peters filter, and by 31.12% over ALTRA.

**Figure 7:**
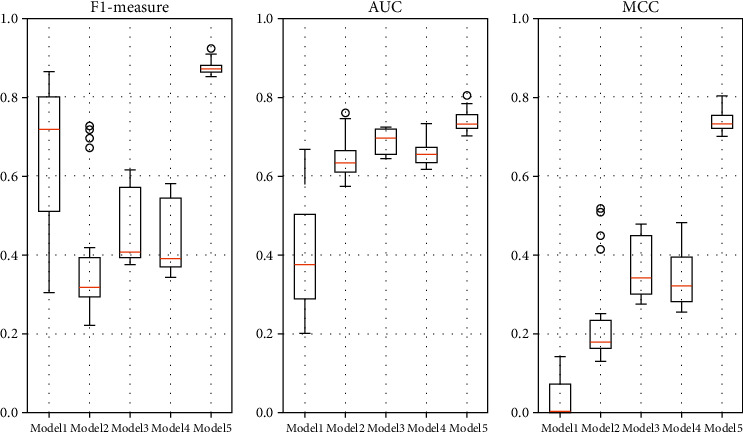
Boxplot of F1-measure, AUC, MCC of ALTRA (Model 1), TCA (Model 2), Peters filter (Model 3), Burak filter (Model 4), and TFIA (Model 5).

Figure 7 is the boxplot of F1-measure, AUC, MCC of ALTRA (Model 1), TCA (Model 2), Peters filter (Model 3), Burak filter (Model 4), and TFIA (Model 5). From Figure 7, we can see that the method in this paper has smaller boxes and shorter endline lengths relative to the other methods, indicating that TFIA performs more consistently on the datasets. Although ALTRA performs better than TCA, Burak filter, and Peters filter on the average F1-measure, the fluctuation of ALTRA is larger. Therefore, we can conclude that the method proposed in this paper possesses good stability and prediction effect on the AEEEM database compared to these methods.

### 5.5. Threats to Validity

In this paper, the threats to validity are mainly divided into internal and external validity.

The internal validity comes from the setting of classifier parameters. In this paper, we set the parameters defaulted by the references as well as the tool of WEKA, which may lead to some differences in the classification results. To mitigate this difference, we use the same default parameters provided by WEKA for the classification phase of our proposed method.

The external validity mainly comes from the dataset used. We use five project datasets from the publicly available AEEEM database and combine 20 source-target project pairs for our experiments. If other data sources are selected, especially those from real-world engineering datasets, different experimental results may be obtained. The attempt on more scenarios will be included in our future research plan.

## 6. Conclusion and Future Work

To tackle the problems of class imbalance and different data distribution among projects in cross-project defect prediction (CcrossPDP) which is the typical application of pattern recognition, we propose a model based on two-phase feature importance amplification (TFIA) in this paper. In the domain adaptation phase, TFIA reduces the differences in data distribution between the source and target projects by adding a certain percentage of samples from the target project to the source project. Meanwhile, correlation-based feature selection (CFS) with a greedy best-first search strategy for feature selection is utilized to obtain a feature subset with a weak feature-feature correlation and a strong feature-class correlation. The source and target project datasets filtered by this feature subset are input to the following classification phase. As the tree-based classifiers are sensitive to features with high feature-class correlation, Random Forest, the integrated method based on Decision Tree for pattern recognition, functions in the classification phase. Multiple decision trees can amplify the importance of features and help each other to improve the performance of the whole prediction model.

We conducted four experiments to validate TFIA on 20 project pairs from the widely adopted AEEEM dataset. The first experiment demonstrates that the domain adaptation approach we designed is effective and has a significant improvement on the overall model performance. The second experiment demonstrates that Random Forest outperforms other classifiers on the dataset processed by our domain adaptation approach. The third experiment analyses the influence of the proportion of samples added from the target projects on the model. The experiment proves that adding 40% of the target item data makes great improvement on the model performance considering the practical application scenarios. In the fourth experiment, we compare TFIA with four other classical research methods, and the experimental result proves that TFIA provides better and more stable performance.

In this paper, our method has been proved to have an improvement effect on CPDP, but there are still some parts that need to be further studied. We will follow two aspects in our future research. The first is to validate the effectiveness of TFIA on other datasets and make it more scalable and robust since our experiments use the subprojects from the same root project. Secondly, since the parameters used in this paper are defaulted, which have a large impact on the prediction model, we will seek to find the effect of adjusting parameters on the model performance. We will also look for the most suitable parameters and the methods that can automatically adjust the parameters according to the real application scenarios.

## Figures and Tables

**Algorithm 1:**
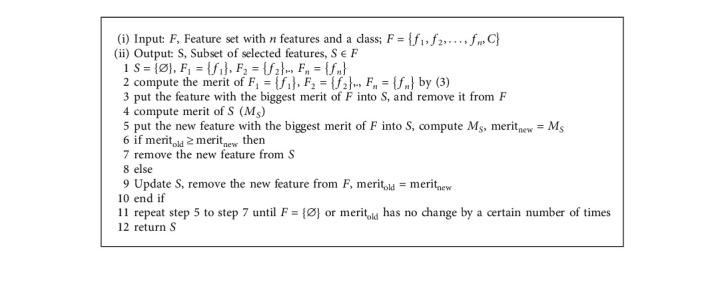
CFS.

**Algorithm 2:**
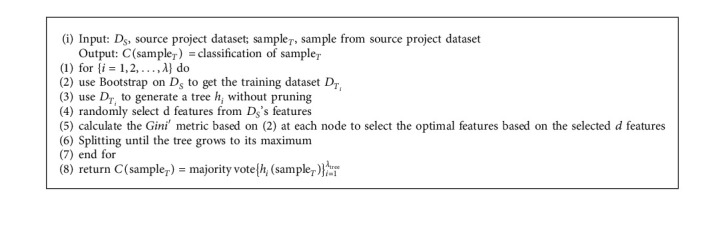
Random Forest.

**Table 1:** Details of AEEEM database.

Projects	Types of projects	Modules	Defective modules	Defective ratio (%)
EQ	OSGI framework	325	129	39.692
JDT	Development	997	206	20.662
LC	Text search engine library	399	64	16.040
ML	Task management	1862	245	13.158
PDE	Development	1492	209	14.008

**Table 2:** F1-measure, AUC, and MCC of model with filtering (Model 1) and model without filtering (Model 2).

	F1-measure		AUC		MCC	
	Model 1	Model 2	Model 1	Model 2	Model 1	Model 2
EQ-JDT	0.415	**0.790**	0.632	**0.873**	0.232	**0.735**
EQ-LC	0.300	**0.761**	0.726	**0.903**	0.265	**0.738**
EQ-ML	0.242	**0.742**	0.545	**0.875**	0.061	**0.702**
EQ-PDE	0.291	**0.776**	0.600	**0.864**	0.146	**0.740**
JDT-EQ	0.276	**0.833**	0.576	**0.862**	0.291	**0.719**
JDT-LC	0.322	**0.752**	0.602	**0.875**	0.330	**0.726**
JDT-ML	0.283	**0.745**	0.584	**0.883**	0.229	**0.707**
JDT-PDE	0.247	**0.761**	0.569	**0.866**	0.233	**0.721**
LC-EQ	0.196	**0.831**	0.554	**0.862**	0.261	**0.710**
LC-JDT	0.592	**0.816**	0.727	**0.911**	0.516	**0.768**
LC-ML	0.347	**0.765**	0.626	**0.855**	0.243	**0.732**
LC-PDE	0.256	**0.787**	0.572	**0.866**	0.234	**0.755**
ML-EQ	0.183	**0.855**	0.550	**0.886**	0.251	**0.755**
ML-JDT	0.396	**0.787**	0.623	**0.883**	0.397	**0.730**
ML-LC	0.219	**0.824**	0.562	**0.925**	0.316	**0.806**
ML-PDE	0.217	**0.763**	0.559	**0.865**	0.229	**0.724**
PDE-EQ	0.238	**0.873**	0.560	**0.897**	0.232	**0.785**
PDE-JDT	0.477	**0.806**	0.666	**0.881**	0.366	**0.754**
PDE-LC	0.372	**0.784**	0.620	**0.873**	0.397	**0.763**
PDE-ML	0.292	**0.754**	0.594	**0.853**	0.178	**0.718**
**Average**	0.308	**0.790**	0.602	**0.878**	0.270	**0.739**

The values in bold are results with the best performance of each instance.

**Table 3:** F1-measure, AUC, and MCC of models with different classifications, including Naive Bayes (NB), Logistic Regression (LR), Decision Tree (DT), Support Vector Classification (SVC), and Random Forest (RF).

	F1-measure					AUC					MCC				
	LR	DT	NB	SVC	RF	LR	DT	NB	SVC	RF	LR	DT	NB	SVC	RF
EQ-JDT	0.594	0.729	0.560	0.571	**0.790**	0.758	0.852	0.713	0.727	**0.873**	0.479	0.655	0.467	0.465	**0.735**
EQ-LC	0.435	0.619	0.404	0.224	**0.761**	0.675	0.889	0.654	0.575	**0.903**	0.387	0.601	0.361	0.136	**0.738**
EQ-ML	0.375	0.672	0.333	0.376	**0.742**	0.647	0.866	0.611	0.637	**0.875**	0.271	0.627	0.253	0.288	**0.702**
EQ-PDE	0.379	0.660	0.361	0.386	**0.776**	0.664	0.851	0.621	0.667	**0.864**	0.259	0.607	0.283	0.269	**0.740**
JDT-EQ	0.595	0.829	0.492	0.459	**0.833**	0.680	0.857	0.644	0.632	**0.862**	0.379	**0.727**	0.358	0.347	0.719
JDT-LC	0.362	0.573	0.373	0.337	**0.752**	0.694	0.837	0.672	0.687	**0.875**	0.293	0.539	0.303	0.266	**0.726**
JDT-ML	0.376	0.637	0.361	0.418	**0.745**	0.644	0.854	0.632	0.679	**0.883**	0.276	0.589	0.265	0.320	**0.707**
JDT-PDE	0.373	0.651	0.356	0.381	**0.761**	0.668	0.841	0.619	0.679	**0.866**	0.253	0.595	0.279	0.265	**0.721**
LC-EQ	0.672	0.806	0.578	0.729	**0.831**	0.727	0.839	0.678	0.773	**0.862**	0.453	0.665	0.389	0.542	**0.710**
LC-JDT	0.575	0.721	0.585	0.525	**0.816**	0.755	0.860	0.743	0.730	**0.911**	0.451	0.648	0.473	0.384	**0.768**
LC-ML	0.393	0.655	0.350	0.406	**0.765**	0.647	0.843	0.620	0.659	**0.855**	0.307	0.604	0.272	0.314	**0.732**
LC-PDE	0.394	0.660	0.367	0.378	**0.787**	0.675	0.843	0.628	0.665	**0.866**	0.279	0.605	0.278	0.258	**0.755**
ML-EQ	0.696	0.826	0.560	0.632	**0.855**	0.730	0.857	0.674	0.687	**0.886**	0.455	0.703	0.397	0.369	**0.755**
ML-JDT	0.567	0.725	0.580	0.539	**0.787**	0.748	0.855	0.728	0.742	**0.883**	0.440	0.650	0.483	0.404	**0.730**
ML-LC	0.343	0.597	0.422	0.338	**0.824**	0.686	0.889	0.681	0.656	**0.925**	0.273	0.583	0.363	0.261	**0.806**
ML-PDE	0.401	0.653	0.381	0.395	**0.763**	0.684	0.837	0.635	0.680	**0.865**	0.288	0.597	0.295	0.281	**0.724**
PDE-EQ	0.640	0.791	0.497	0.525	**0.873**	0.704	0.826	0.645	0.638	**0.897**	0.410	0.643	0.357	0.299	**0.785**
PDE-JDT	0.585	0.701	0.602	0.521	**0.806**	0.746	0.834	0.752	0.733	**0.881**	0.470	0.619	0.495	0.382	**0.754**
PDE-LC	0.382	0.584	0.438	0.362	**0.784**	0.702	0.857	0.690	0.639	**0.873**	0.315	0.557	0.380	0.306	**0.763**
PDE-ML	0.385	0.629	0.312	0.406	**0.754**	0.646	0.831	0.600	0.666	**0.853**	0.291	0.573	0.232	0.308	**0.718**
**Average**	0.476	0.686	0.446	0.445	**0.790**	0.694	0.851	0.662	0.678	**0.878**	0.351	0.619	0.349	0.323	**0.739**

The data in bold shows the classification with the best perfomance in each set of experiments.

**Table 4:** Average F1-measure, AUC, and MCC of TFIA using different proportions samples from target project datasets added to source project datasets.

Proportion (%)	Average F1-measure	Average AUC	Average MCC
0	0.352	0.609	0.221
10	0.458	0.668	0.350
20	0.575	0.736	0.475
30	0.683	0.804	0.607
40	0.790	0.878	0.739
50	0.866	0.928	0.832
60	0.921	0.963	0.902
70	0.953	0.981	0.942
80	0.969	0.990	0.962
90	0.980	0.995	0.976

**Table 5:** F1-measure, AUC, and MCC of ALTRA (Model 1), TCA (Model 2), Peters filter (Model 3), Burak filter (Model 4), and TFIA (Model 5).

	F1-measure					AUC					MCC				
	Model 1	Model 2	Model 3	Model 4	Model 5	Model 1	Model 2	Model 3	Model 4	Model 5	Model 1	Model 2	Model 3	Model 4	Model 5
EQ-JDT	0.448	0.419	0.574	0.552	0.873	0.266	0.638	0.729	0.707	0.735	-0.079	0.244	0.467	0.462	0.735
EQ-LC	0.449	0.220	0.404	0.384	0.903	0.286	0.613	0.719	0.658	0.738	-0.064	0.131	0.342	0.323	0.733
EQ-ML	0.304	0.320	0.376	0.353	0.875	0.253	0.656	0.646	0.623	0.702	-0.275	0.211	0.274	0.267	0.699
EQ-PDE	0.415	0.306	0.407	0.371	0.864	0.203	0.624	0.657	0.635	0.740	-0.233	0.180	0.309	0.269	0.740
JDT-EQ	0.526	0.727	0.598	0.459	0.862	0.388	0.760	0.694	0.632	0.719	-0.109	0.517	0.432	0.347	0.719
JDT-LC	0.704	0.235	0.405	0.377	0.875	0.271	0.647	0.705	0.665	0.726	0.062	0.172	0.340	0.309	0.725
JDT-ML	0.725	0.319	0.375	0.361	0.883	0.605	0.659	0.647	0.632	0.707	0.142	0.216	0.271	0.265	0.702
JDT-PDE	0.713	0.289	0.407	0.388	0.866	0.668	0.599	0.661	0.649	0.721	0.051	0.151	0.305	0.281	0.721
LC-EQ	0.465	0.673	0.588	0.566	0.862	0.297	0.708	0.683	0.674	0.710	-0.152	0.410	0.397	0.389	0.701
LC-JDT	0.868	0.379	0.567	0.580	0.911	0.441	0.576	0.725	0.734	0.768	0.091	0.161	0.458	0.472	0.762
LC-ML	0.862	0.322	0.378	0.348	0.855	0.341	0.665	0.647	0.619	0.732	-0.017	0.224	0.277	0.265	0.731
LC-PDE	0.792	0.298	0.397	0.394	0.866	0.605	0.612	0.657	0.652	0.755	0.078	0.164	0.290	0.289	0.754
ML-EQ	0.710	0.719	0.618	0.543	0.886	0.579	0.746	0.705	0.668	0.755	0.115	0.507	0.446	0.400	0.740
ML-JDT	0.751	0.376	0.568	0.567	0.883	0.309	0.572	0.724	0.721	0.730	-0.099	0.149	0.461	0.464	0.727
ML-LC	0.808	0.224	0.374	0.406	0.925	0.540	0.634	0.703	0.682	0.806	0.019	0.162	0.308	0.341	0.804
ML-PDE	0.806	0.294	0.408	0.390	0.865	0.287	0.606	0.664	0.649	0.724	-0.018	0.158	0.302	0.286	0.724
PDE-EQ	0.644	0.695	0.614	0.513	0.897	0.373	0.721	0.705	0.657	0.785	-0.174	0.450	0.453	0.390	0.784
PDE-JDT	0.800	0.384	0.571	0.582	0.881	0.388	0.586	0.721	0.731	0.754	0.077	0.169	0.476	0.481	0.754
PDE-LC	0.800	0.225	0.402	0.370	0.873	0.375	0.636	0.727	0.659	0.763	0.068	0.163	0.343	0.303	0.763
PDE-ML	0.800	0.325	0.384	0.342	0.853	0.491	0.669	0.654	0.618	0.718	0.068	0.230	0.281	0.254	0.718
Average	0.670	0.387	0.471	0.442	0.878	0.398	0.646	0.689	0.663	0.739	-0.022	0.238	0.362	0.343	0.737

## Data Availability

A publicly available dataset is used for this study (AEEEM).
